# Diagnostic Utility of CSF Tau and A**β**_42_ in Dementia: A Meta-Analysis

**DOI:** 10.4061/2011/503293

**Published:** 2011-12-04

**Authors:** Rachna Agarwal, Chandra Bhushan Tripathi

**Affiliations:** ^1^Department of Neurochemistry, Institute of Human Behaviour & Allied Sciences, Dilshad Garden, Delhi 110095, India; ^2^Department of Biostatics, Institute of Human Behaviour & Allied Sciences, Delhi 110095, India

## Abstract

CSF tau and A**β**42 are considered as important markers to diagnose Alzheimer's disease in early stages. Hence, it is important to assess their status in different types of dementia. The main objective of this study was to assess whether these CSF biomarkers can be used to make the differential diagnosis of AD. In the present study, articles published from 1998 till 2009 were taken and meta-analysis was performed to clarify the consistency in trends of biomarkers- CSF tau and A**β**_42_ in AD and other dementias and whether the same can be used as diagnostic biomarkers for its early diagnosis. 11 out of 60 for CSF tau and 07 out of 40 for CSF A**β**_42_, dementia case-control studies were selected for final analysis. Descriptive statistics shows that median effect size (raw mean difference) of CSF tau was 429 pg/mL (range: 32 to 910 pg/mL) in AD whereas in Dementia due to other causes (DOC) studies it was 69 pg/mL (range: −53 to 518 pg/mL). Similarly the median effect size of CSF A**β**_42_ levels was −442 pg/mL (range: −652 to −41.200 pg/mL) whereas in DOC studies it was −193 pg/mL (range: −356 to −33 pg/mL).

## 1. Introduction

With the increase in life expectancy, Alzheimer's disease, considered as disease of aging population, has become a major public health problem adding burden to societal costs each year for chronic care and lost productivity in developed and developing countries [1]. Presently, diagnosis of AD is primarily based on the exclusion of other causes of dementia on clinical trials. However, it is not of much help due to overlapping of clinical features of AD with other dementias in early stages. Attempts have been made in the last 4-5 decades to develop and validate specific biological markers which are able to detect the fundamental neuropathological changes occurring in AD in its early stage with high sensitivity (≥80%) and distinguish it from other dementias [2]. Based on these studies, the combination of total tau and amyloid *β*_42_ (A*β*_42_) was identified as being among the most promising and informative AD markers to be of use in early diagnosis and as surrogate biomarkers in CSF [2–4]. Increased levels of tau in CSF have been suggested to reflect neuronal and axonal degeneration [5] whereas reduced CSF levels of A*β*_42_ might reflect extracellular accumulation of A*β*_42_ into insoluble senile plaques in the AD brain [6, 7]. However, high CSF concentration of total tau has also been reported in mild cognitive impairment [8], as well as in vascular dementia (VaD) [2, 9–11]. The comorbidity of AD and other dementias can further generate problems. Though studies have reported highly accurate differentiation between AD and normal controls with high sensitivity (50–94%) and specificity (83–100%) [12], CSF-based differentiation of AD with VaD remains a challenge with specificity of 48% only [13]. Also, AD can be associated with other neurodegenerative diseases like Lewy body disease and progressive supranuclear palsy. 

In view of the necessity of identifying biomarkers for differentiation of AD from other dementia disorders, the combination of CSF tau and A*β*_42_ has been advocated as diagnostic marker. However, before the diagnostic utility of CSF tau and A*β*_42_ concentration for diagnosis of AD can be established, it is crucial to assess whether the effect sizes of the published studies reported in the last fifteen years show consistent trends in their levels or not in AD cases as compared to controls. The consistency in effect size of tau and A*β*_42_ levels of these studies was explored by advanced statistical method meta-analysis with three main goals: (i) to test whether the results of studies are homogeneous, (ii) to obtain a global index about the effect magnitude of the studied relation, joined to a confidence interval and its significance, and (iii) to identify possible variables or characteristics moderating the results obtained if there is heterogeneity among studies [14]. In the present study, studies conducted from 1995 till 2009 were taken and meta-analysis was performed to clarify the consistency in trends of biomarkers CSF tau and A*β*_42_ in different types of dementias and whether the same can be used as diagnostic biomarkers for early diagnosis of AD.

## 2. Materials and Methods

### 2.1. Search Strategy

Meta-analysis was performed for CSF tau and A*β*_42_ levels, by calculating and combining the effect sizes (raw mean difference), their standard error, and 95% confidence interval after extracting mean, standard deviation, and sample size from 11 CSF studies. 11 studies out of 60 studies for CSF tau levels and 7 studies out of 41 studies were selected for CSF A*β*_42_ levels in dementias from 1996 to 2009 using keywords CSF biomarkers in Alzheimer's disease, tau levels in Alzheimer's disease, and A*β*_42_ levels in Alzheimer's disease.

### 2.2. Exclusion Criteria

Studies were excluded if they were not in English language, did not provide data for controls, did not mention the diagnostic criteria for AD, and did not report the SD of the mean CSF tau and A*β*_42_ level or their units were not in pg/mL. Also, studies where tau and A*β*_42_ were measured by methods other than sandwich enzyme linked immunosorbant assay were excluded. Only those studies were undertaken in which CSF tau and A*β*_42_ levels were measured in AD and other dementias (vascular dementia, Parkinson's disease-associated dementia, and Lewy body dementia) in controls using Innotest kit from Innogenetics, Belgium. All the studies included had interday and intraday variation less than 10%. Other-dementias group was named dementia due to other causes (DOC). All the values were expressed in pg/mL.

### 2.3. Statistical Analysis

The raw mean difference (unstandardized) was used for calculating the effect size of each study. The heterogeneity in effect size of both parameters (tau and A*β*_42_), which may occur from study to study and is one of the serious issues of any meta-analysis, was explored with the help of three statistical tools: (1) test of significance (Q statistics), (2) between-studies variance (*T* ^2^), and (3) degree of heterogeneity (*I* ^2^). An appropriate model (fixed effect versus random effect model) for getting the pooled effect size was decided after evaluating the heterogeneity in effect size of all the included studies. 

The graphical method (Forest plot) has been applied for studying the variability between the effect sizes of individual studies. In Forest plot, each study effect size and respective confidence interval (CI) were plotted on one set of axis along with pooled estimate of effect size, together with its CI. 

All the statistical analysis was done using the Meta-analysis report software (Version: Beta3.13) downloaded from the web site on 05.10.10.

## 3. Results

As CSF tau and A*β*_42_ levels have emerged as two biomarkers for early diagnosis of dementia, in this paper, meta-analysis of published studies for CSF tau and A*β*_42_ level has been carried out to establish the role of combination of two biomarkers in Alzheimer's disease and dementia due to other causes (DOC).

### 3.1. Meta-Analysis of CSF Tau Level in Dementia

A meta-analysis was performed in 11 published studies of CSF tau in AD and DOC, selected from total of 60 studies. Out of total published studies included in meta-analysis of CSF tau levels in DOC 5 published studies were of vascular dementia (VaD) [7, 18, 22–24], 2 of Parkinson's disease-associated dementia (PDD) [15, 20], 1 of mixed dementia [16], and the rest were non-AD dementia (non-ADD) [17, 19]. Out of 11 studies, only two studies in AD [15, 23] and one study in DOC [23] were having matched controls.

Table 1 shows that details of 11 included studies along with mean, SD, and sample size (N) of CSF tau level for cases and controls. Effect size (raw mean difference), standard error, 95% confidence interval and weight for each included study were calculated. None of studies show effect size zero in both group (Alzheimer's disease and DOC).

Table 2 shows that median sample size (case + control) in 11 Alzheimer's disease studies of CSF tau level was 67 (range: 30–142) whereas it was 49 (range: 26–134) in dementia-due-to-other-causes studies. The median effect size (raw mean difference) of tau level in Alzheimer's disease studies was 429 pg/mL (range: 32 to 910 pg/mL) whereas in dementia-due-to-other-causes studies it was 69 pg/mL (range: −53 to 518 pg/mL). In Alzheimer's disease, CSF tau level is quite high as compared to DOC. 

Table 3 shows the estimated pooled effect size of CSF tau level and its 95% confidence interval (CI) of 11 included studies for both groups. Between-studies variance, degree of heterogeneity with 95% CI, Q statistics, degree of freedom, and *P* value were also shown in the same tables. From Table 3, it can be seen that between-studies variance, degree of heterogeneity, and Q statistics all three approaches clearly indicate that variability in CSF tau estimates across the studies was too high and random effect model for pooling of CSF tau estimates is the only choice. After applying random effect model for pooling of estimates, the pooled estimate of CSF tau in Alzheimer's disease studies was 414.073 pg/mL (CI: 237.170–590.975) while it was quiet low as 83.500 pg/mL (CI: 36.406–130.595) in dementia-due-to-other-causes studies. 

**Figure 1:**
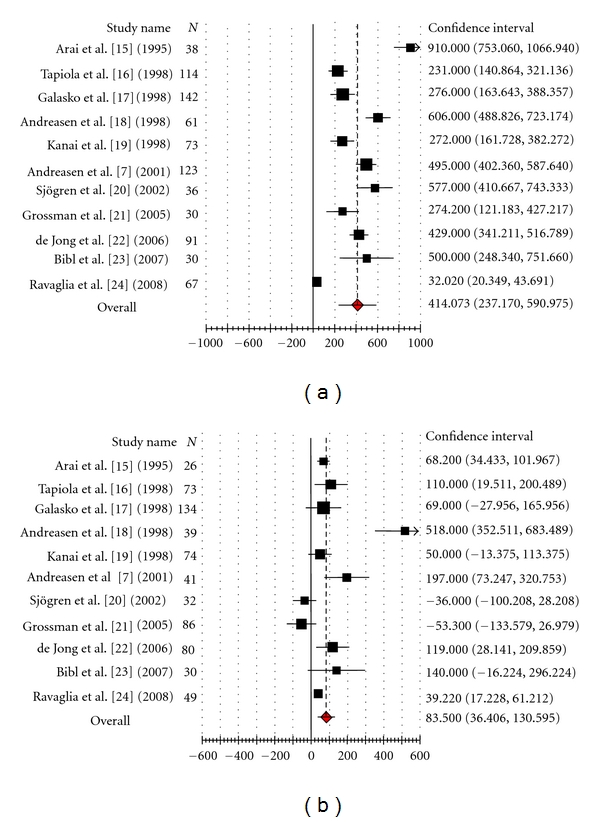
(a) CSF tau level in Alzheimer's disease. (b) CSF tau level in dementia due to other causes.

Forest plot (Figure 1(a)) shows that out of 11 Alzheimer's disease studies, effect size (raw mean difference) of 6 studies [13, 15, 18, 20, 22, 23] was more than pooled estimate of effect size, and in 5 studies [16, 17, 19, 21, 24], it was below the pooled effect size, whereas out of 11 dementia-due-to-other-causes studies (Figure 1(b)), 5 studies were having individual effect size more than pooled estimate of effect size [13, 16, 18, 22, 23], while in 6 studies, individual effect size was below the pooled effect size [15, 17, 19–21, 24]. 

### 3.2. Meta-Analysis of CSF Amyloid *β*_42_ Level in Dementia

A meta-analysis was performed in 7 published studies of CSF tau in AD and DOC, selected from total of 41 studies. Out of total published studies included in meta-analysis of CSF A*β*_42_ levels in DOC 3 published studies were of vascular dementia (VaD) [13, 22, 23], 1 of Parkinson's disease-associated dementia (PDD) [20], 1 of semantic dementia [21], and the rest were non-AD dementia (non-ADD) [17, 19].

Table 4 shows that details of 7 included studies along with mean, SD, and sample size (N) of CSF A*β*_42_ level for cases and controls. Effect size (raw mean difference), standard error, 95% confidence interval, and weight for each included study were calculated. None of studies show effect size zero in both group (Alzheimer's disease and dementia due to other causes).

Table 5 shows that median sample size (case + control) in 7 Alzheimer's disease studies of CSF A*β*_42_ level was 73 (range: 30–142) whereas it was 41 (range: 26–134) in dementia-due-to-other causes studies. The median effect size (raw mean difference) of A*β*_42_ level in Alzheimer's disease studies was −442 pg/mL (range: −652 to −41.200 pg/mL) whereas in dementia-due-to-other causes studies it was −193 pg/mL (range: −356 to −33 pg/mL). In Alzheimer's disease, median CSF A*β*_42_ level is quite low as compared to DOC. 

Table 6 shows the pooled effect size of CSF A*β*_42_ level and its 95% confidence interval (CI) of 7 included studies. Between-studies variance, degree of heterogeneity with 95% CI, Q statistics, degree of freedom, and *P* value were also shown in the same tables. From Table 6, it can be seen that between-studies variance, degree of heterogeneity, and Q statistics all three approaches clearly indicate that variability in CSF A*β*_42_ estimates across the studies was too high and random effect model for pooling of CSF A*β*_42_ estimates is the only choice. After applying random effect model for pooling of estimates, the pooled estimate of CSF A*β*_42_ in Alzheimer's disease studies was −363.926 pg/mL (CI: −542.007–−185.845) while it was quiet high as −170.743 pg/mL (CI: −256.912–−84.574) in DOC studies. 

**Figure 2:**
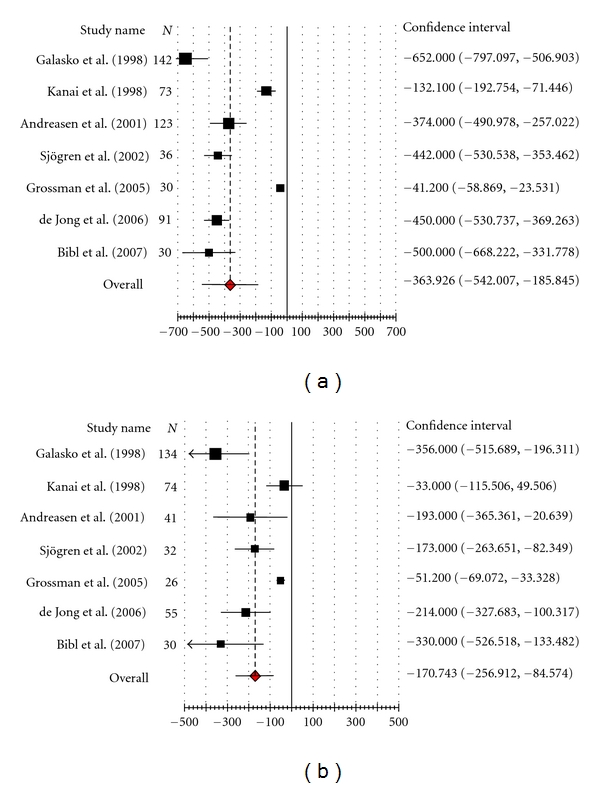
(a) CSF Amyloid *β*(1–42) level in Alzheimer's disease. (b) CSF amyloid *β*(1–42) level in dementia due to other causes.

Forest plot (Figure 2(a)) shows that out of 7 Alzheimer's disease studies, effect size (raw mean difference) of 2 studies was more than pooled estimate of effect size [19, 21] and, in 05 studies, it was less than the pooled effect size [13, 17, 20, 22, 23]. Of 7 DOC studies (Figure 2(b)), 02 studies were also having individual effect size more than pooled estimate of effect size [19, 21] while in 05 studies individual effect size was below the pooled effect size [13, 17, 20, 22, 23]. 

Funnel plots showed clear existence of publication bias in both groups.

## 4. Discussion

Diagnostic markers for AD have been sought for many years for its early diagnosis and differentiating it from other types of dementias. Distinguishing between the two most common forms of dementias, AD and VaD, is one of the most challenging differential diagnoses in geriatrics OPD and is very crucial also because the therapeutic strategies are very different. With the development of cholinergic-based treatments for AD, there is great emphasis on the need for its early and accurate diagnosis to allow initiation of therapy when it will be of most benefit to the patients [17]. CSF biomarkers tau and A*β*_42_ combined together have emerged as such diagnostic markers for early diagnosis of AD. Although combination of CSF tau and A*β*_42_ yields a highly accurate differentiation between AD and controls, CSF-based differentiation of AD from other dementias especially VaD remains highly challenging as it has low specificity for the same [13].

### 4.1. Meta-Analysis of CSF Tau Level

A meta-analysis performed in 11 published studies of CSF tau in different types of dementias showed high levels of tau in AD in all the studies whereas 2 studies showed low levels in DOC as compared to controls. A number of studies have found a significant increase in CSF tau levels in AD and DOC as compared with normal ageing [25–27]. 

Table 2 shows that raw mean difference of CSF tau levels in AD group was much higher than that in DOC group signifying that levels of tau in CSF in AD cases rise much higher than in DOC. This finding supports the studies showing much higher sensitivity and specificity of CSF tau levels in distinguishing the AD patients from control subjects as compared to non-AD dementias [16] and VaD [13].

The assessment of the heterogeneity in meta-analysis is a crucial issue because the presence versus absence of true heterogeneity (between-studies variability) can affect the choice of statistical model. There can be two sources of variability leading to heterogeneity in a set of studies in a meta-analysis. First, variability due to sampling error (within-study variability) is always present in a meta-analysis because every single study uses different samples. The other source of variation is due to heterogeneity among the population effect sizes estimated by the individual studies (between-studies variability). It may be due to variation in the characteristics of the samples, treatment and the design quality of the study [27]. In the present study, three statistical methods were applied to explore the true heterogeneity. They strongly recommend the presence of heterogeneity in a set of 11 tau studies (Table 3). The test of significance (*P* = 0.000) suggests the presence of true heterogeneity among the effect size of all 11 studies. The magnitude of the true heterogeneity due to between studies variance (*T* ^2^ = 85013.958 for AD versus controls, & 4343.165 for DOC versus controls) was much higher and contributed to 97.9% in AD versus control and 82.4% in DOC versus control (degree of heterogeneity *I* ^2^) in total heterogeneity. All the studies selected, fulfilling the defined inclusion criteria, report a significant difference in CSF tau levels in AD and DOC participants versus controls. Despite the uniform pattern of CSF tau level changes in AD and DOC as compared to control, there was wide range of effect size among 11 articles under study in AD group (32.02–910.00 pg/mL) as compared to DOC group (−53.30–−518.00 pg/mL). There were large differences in baseline levels across studies; 6 studies' in AD versus control [13, 15, 18, 20, 22, 23] and 05 studies in DOC versus control have effect size more than pooled estimate [13, 16, 18, 22, 23], signify that dementia patients having higher CSF tau levels than control. However, the increase in CSF tau levels in AD was much higher than in DOC as compared to control in most studies. Only Ravaglia et al. 2008 [24] found low effect size (raw mean difference) of tau levels in DOC as compared to AD.

A number of confounding factors are responsible for the heterogeneity and wide range of CI of effect size found in the studies undergoing meta-analysis. First, very few studies could be included for meta-analysis as per the inclusion criteria in the present study; second, except one study [17] all of the studies had very low sample size for DOC group. Also most of the studies were not age-, sex-matched, and of 11 AD studies, 7 studies were having more number of cases than controls [13, 16–18, 20–22] while in 4 studies the number of control was either equal or more than the number of cases [15, 19, 23, 24]. Similarly, in DOC group, 6 studies were having more number of cases than controls [13, 16–18, 21, 22]. There are inconsistent findings reported showing discrepancies in the effect of these on CSF tau levels in AD versus controls. Some investigators have reported increase of tau with age [15, 19] and a negative correlation with sex [17] with a tendency for females to have higher tau levels than males whereas others found tau levels unaffected by age, age at onset, and AD duration, severity, and rate of progression and equally increased in early and late disease, in mild and severe disease [17, 19, 21]. Another confounding factor is variation in freezer shelf life introducing variation in storage conditions for CSF.

### 4.2. Meta-Analysis of Amyloid *β*_42_ Level

All the published studies undertaken in the present meta-analysis showed low CSF A*β*_42_ levels in AD and DOC as compared to controls. However, one study effect size had wider confidence interval with upper limit more than zero in both AD and DOC [21]. Table 5 shows that median effect size of A*β*_42_ levels in CSF in AD is −442 pg/mL as compared to −193 pg/mL in DOC which indicates that decrease in CSF A*β*_42_ in AD is much higher than in DOC group as compared to controls. It is supported by the studies done in last two decades reporting low CSF A*β*_42_ levels in early stages of AD with high degree of sensitivity and specificity [25] as compared to other dementias-LBD [13] and VaD [13].

Out of 7 studies undertaken, 5 studies in AD versus control group had more number of cases than controls [13, 17, 20–22] while the other 2 studies had either equal number of controls or less than cases [19, 23], whereas in DOC versus control group, only 2 studies had more number of cases than controls [13, 17] and the other 5 studies had either more or equal number of controls than cases [19–23]. 

Same three statistical techniques as have been applied in CSF tau studies (mentioned above) were used to determine the true heterogeneity (between-studies variability) in effect size of 7 included studies (Table 6). The test of significance (*P* = 0.000) rejects the null hypothesis of similar effect size in all the studies, and the estimated between-studies variance (between studies variance *T* ^2^ = 54859.742 for AD versus controls and 9755.039 for DOC versus controls) was very much high and between-studies variance contributes the most in total heterogeneity (degree of heterogeneity *I* ^2^ = 97.8, CI: 96.8%–98.5% in AD and degree of heterogeneity *I* ^2^ = 83.7, CI: 67.9%–91.7% in DOC). The heterogeneity in effect size of these 07 published studies could be due to different population, small sample size and lack of standardization of assay methods [26]. Though in AD group all 7 studies showed significant reductions in CSF A*β*_42_ levels as compared to control participants, the result of the present study was unequivocal and the range of effect size of 7 studies was quite large (−652 to −41.2 pg/mL). However, the fall in DOC group was much lower than in AD when compared to the control with reduced range of effect size of 7 studies undertaken (−33 to −356 pg/mL). Due to wide range of effect size of these studies, 2 studies effect size was more [19, 21] and that of 5 studies was less than the pooled estimate [17, 20, 22, 23] whereas 1 study [13] was on the vertical line passing through the pooled effect size. Similarly, in DOC group, 2 studies effect size was more [19, 21] and that of 5 studies was less than the pooled estimate [13, 17, 22, 23] whereas 1 study [20] was on the vertical line passing through the pooled effect size.

It has been a challenging task to explain the wide variation in results in the different laboratories due to technical problems in the studies included for studying the diagnostic utility of CSF biomarkers tau and A*β*_42_ in dementia. To overcome this, sensitivity analysis was conducted to explore the sensitivity level of each study. It was observed that there was not much difference in overall effect size on exclusion of one study at a time. Another major problem with this meta-analysis study is the presence of “publication bias.” Several lines of evidence show [27] that studies that report relatively high effect sizes are more likely to be published than studies that report lower effect sizes. In the present study, Funnel plot analysis was done which showed the presence of publication bias for both parameters in AD as well as DOC group supporting the hypothesis that any bias in the literature is likely to be reflected in the meta-analysis study as only statistically significant results are more likely to be published.

### 4.3. Limitation

During the present meta-analysis, a number of factors was observed which could be causing the limitation in developing hypothesis/coming to conclusion regarding using CSF tau and A*β*_42_ as diagnostic markers in differential diagnosis of AD from dementias due to other causes. One limitation encountered during the present study was simply the amount of effort and expertise meta-analysis takes. Also, lack of complete information in some studies and high level of heterogeneity in individual study effect size for both parameters (tau & A*β*_42_) added to the limitation. Variation in CSF tau and A*β*_42_ levels in AD, DOC, and controls may be due to the variability introduced during sample collection and storage like using glass or polystyrene tubes^18^ than polypropylene tubes, prolonged storage of CSF samples in frozen state or repeated freezing and thawing of CSF, errors introduced due to inadequate study plan like small sample size, less number of controls taken in the study as compared to cases, or case and controls being not age and sex matched [24].

## 5. Conclusion

Based on our findings of the present meta-analysis, it can be concluded that the combination of high tau and low A*β*_42_ is highly specific for AD and might be useful in screening out the suspected cases of AD, from other types of dementia. However, due to the limited number of studies having large number of sample size with age- and sex-matched samples in control and cases available, the use of these biomarkers has to be verified in further prospective studies.

## Figures and Tables

**Table 1:** Effect size, its 95% CI, and % weight calculations for each selected study of CSF tau in Alzheimer's disease and dementia due to other causes.

S. no.	Author's name and year	Country where the studies were conducted	Tau level (pg/mL) (AD and control)						Effect size (pg/mL)		95% CI	Weight	Tau level (pg/mL) (DOC and control)						Effect size (pg/mL)		95% CI	Weight
			Diseased			Control							Diseased			Control						
			N	M_d_*	SD	N	M_c_**	SD	RMD^+^	SE			N	M_d_*	SD	N	M_c_**	SD	RMD^+^	SE		
1	Arai et al. (1995) [15]	Japan	19	919	349	19	9	4.5	910.00	80.07	753.06–1066.94	0.005	7	77.2	45.5	19	9	4.5	68.20	17.23	34.43–101.97	0.215
2	Tapiola et al. (1998) [16]	Finland	81	524	351	33	293	140	231.00	45.99	140.86–321.14	0.015	40	403	248	33	293	140	110.00	46.17	19.51–200.49	0.030
3	Galasko et al. (1998) [17]	USA	82	663	481	60	387	167	276.00	57.33	163.64–388.36	0.010	74	456	383	60	387	167	69.00	49.47	−27.96–165.96	0.026
4	Andreasen et al. (1998) [18]	Sweden	43	796	382	18	190	57	606.00	59.78	488.83–723.17	0.009	21	708	382	18	190	57	518.00	84.44	352.51–683.49	0.009
5	Kanai et al. (1998) [19]	Japan	32	489	297.5	41	217	128	272.00	56.26	161.73–382.27	0.010	33	267	146	41	217	128	50.00	32.34	−13.38–113.38	0.061
6	Andreasen et al. (2001) [7]	Sweden	105	759	417	18	264	102	495.00	47.27	402.36–587.64	0.014	23	461	280	18	264	102	197.00	63.14	73.25–320.75	0.016
7	Sjögren et al. (2002) [20]	Sweden	19	919	349	17	342	116	577.00	84.87	410.67–743.33	0.004	15	306	65	17	342	116	−36.00	32.76	−100.21–28.21	0.059
8	Grossman et al. (2005) [21]	US	17	534.6	303.5	13	260.4	93.8	274.20	78.07	121.18–427.22	0.005	73	207.1	270.3	13	260.4	93.8	−53.30	40.95	−133.58–26.98	0.038
9	de Jong et al. (2006) [22]	The Netherlands	61	613	326	30	184	89	429.00	44.79	341.21–516.79	0.016	50	303	307	30	184	89	119.00	46.36	28.14–209.86	0.030
10	Bibl et al. (2007) [23]	Germany	15	700	480	15	200	130	500.00	128.40	248.34–751.66	0.002	15	340	280	15	200	130	140.00	79.71	−16.22–296.22	0.010
11	Ravaglia et al. (2008) [24]	Italy	31	56.3	32.7	36	24.28	5.9	32.02	5.96	20.35–43.69	0.909	13	63.5	40.3	36	24.28	5.9	39.22	11.22	17.23–61.21	0.506

*Mean in diseased group, **Mean in control group and raw mean difference.

**Table 2:** Descriptive statistics of CSF tau studies in Alzheimer's disease and dementia due to other causes.

CSF tau level (pg/mL)	Alzheimer's disease		Dementia due to other causes	
	Median	Range	Median	Range
Sample size	67	30–142	49	26–134
Effect size	429	32.020–910	69	−53.300–518

**Table 3:** Pooled estimate of effect size with 95% CI, test of significance and magnitude of heterogeneity of CSF tau studies in Alzheimer's disease and Dementia due to other causes.

Statistics	Alzheimer's disease	Dementia due to other causes
Pooled estimate of CSF tau (pg/mL)	414.073	83.500
95% CI of pooled estimate (pg/mL)	237.170–590.975	36.406–130.595
Between-studies variance (*T* ^2^)	85013.958	4343.165
Degree of heterogeneity (*I* ^2^)	97.9%	82.4%
95% CI of *I* ^2^	97.2%–98.4%	69.7%–89.7%
Q statistics	467.756	56.738
DF	10	10
*P* value	0.000	0.000

**Table 4:** Effect size, its 95% CI, and % weight calculations for each selected study of CSF A*β*_42_ in Alzheimer's disease and dementia due to other causes.

S. no.	Author's name and year	Country where the studies were conducted	A*β*42 level (pg/mL) (AD and control)						Effect size (pg/mL)		95% CI	Weight	A*β*_42_ level (pg/mL) (DOC and control)						Effect size (pg/mL)		95% CI	Weight
			Diseased			Control							Diseased			Control						
			N	M_d_*	SD	N	M_c_**	SD	RMD^+^	SE			N	M_d_*	SD	N	M_c_**	SD	RMD^+^	SE		
1	Galasko et al. (1998) [17]	USA	82	833	379	60	1485	473	−652.000	74.031	−797.10–−506.90	0.012	74	1129	464	60	1485	473	−356.000	81.475	−515.69–−196.31	0.011
2	Kanai et al. (1998) [19]	Japan	32	109.9	73.2	41	242	180	−132.100	30.947	−192.75–−71.45	0.069	33	209	180	41	242	180	−33.000	42.096	−115.51–−49.51	0.041
3	Andreasen et al. (2001) [7]	Sweden	105	523	180	18	897	242	−374.000	59.684	−490.98–−257.02	0.019	23	704	321	18	897	242	−193.000	87.941	−365.36–−20.64	0.009
4	Sjögren et al. (2002) [20]	Sweden	19	411	99	17	853	161	−442.000	45.173	−530.54–−353.46	0.033	15	680	96	17	853	161	−173.000	46.251	−263.65–−82.35	0.034
5	Grossman et al. (2005) [21]	USA	17	54	15.1	13	95.2	29.7	−41.200	9.015	−58.87–−23.53	0.819	13	44	14.1	13	95.2	29.7	−51.200	9.118	−69.07–−33.33	0.876
6	de Jong et al. (2006) [22]	The Netherlands	61	419	128	30	869	207	−450.000	41.193	−530.74–−369.26	0.039	25	655	220	30	869	207	−214.000	58.003	−327.68–−100.327	0.022
7	Bibl et al. (2007) [23]	Germany	15	310	90	15	810	320	−500.000	85.829	−668.22–−331.78	0.009	15	480	220	15	810	320	−330.000	100.266	−526.52–−133.48	0.007

*Mean in diseased group, **Mean in control group and raw mean difference.

**Table 5:** Descriptive statistics of CSF A*β*_42_ studies in Alzheimer's disease and dementia due to other causes.

CSF A*β*42 level (pg/mL)	Alzheimer's disease		Dementia due to other causes	
	Median	Range	Median	Range
Sample size	73	30–142	41	26–134
Effect size	−442	−652–−41.200	−193	−356–−33

**Table 6:** Pooled estimate of effect size with 95% CI, test of significance, and magnitude of heterogeneity of CSF A*β*_42_ studies in Alzheimer's disease and dementia due to other causes.

Statistics	Alzheimer's disease	Dementia due to other causes
Pooled estimate of CSF A*β*_42_ (pg/mL)	−363.926	−170.743
95% CI of pooled estimate (pg/mL)	−542.007–−185.845	−256.912–−84.574
Between-studies variance (*T* ^2^)	54859.742	9755.39
Degree of heterogeneity (*I* ^2^)	97.8%	83.7%
95% CI of *I* ^2^	96.8%–98.5%	69.7%–91.7%
Q statistics	271.051	36.780
DF	6	6
*P* value	0.000	0.000
